# Broadband wavelength tuning of electrically stretchable chiral photonic gel

**DOI:** 10.1515/nanoph-2021-0645

**Published:** 2022-01-04

**Authors:** Seungmin Nam, Dahee Wang, Gyubin Lee, Su Seok Choi

**Affiliations:** Department of Electrical Engineering, Pohang University of Science and Technology (POSTECH), 77 Cheongam-Ro, Nam Gu, Pohang, Gyeongbuk 37673, Republic of Korea

**Keywords:** chiral liquid crystals, chiral photonic gel, chiral photonic-band structures, soft actuator, wavelength tuning

## Abstract

Chiral photonic-band structure provides technical benefits in the form of a self-assembled helical structure and further functional wavelength tunability that exploits helical deformation according to pitch changes. The stopband wavelength control of the chiral photonic-band structure can be obtained by individual electrical methods or mechanical stretching deformation approaches. However, research on combined electric control of stretchable chiral photonic-band wavelength control while ensuring optical stability during the tuning process has remained limited till now. In this study, using the hybrid structure of elastomeric mesogenic chiral photonic gels (CPGs) with an electrically controlled dielectric soft actuator, we report the first observation of electrically stretchable CPGs and their electro-mechano-optical behaviors. The reliable wavelength tuning of a CPG to a broadband wavelength of ∼171 nm changed with high optical stability and repeated wavelength transitions of up to 100 times. Accordingly, for the first time, electrical wavelength tuning method of stretchable chiral liquid crystal photonicband structure was investigated.

## 1 Introduction

A chiral photonic-band structure using liquid crystals can spontaneously self-organize, enabling it to exploit the helicoidal optical rotation of mesogenic molecules, which have an effective birefringence [1]. Accordingly, this chiral photonic-band structure, which employs the birefringent soft matter of liquid crystals, can produce well-organized dielectric periodicity at the nanometer scale and provide a high-quality stopband for electromagnetic wave propagation. This self-organized chiral photoinc structure significantly contrasts the conventional photonic nano-process, which is expensive and must be limited in scalability for practical applications. Owing to these technical benefits, chiral photonic-band structures have attracted growing research interest for various photonic applications, e.g., in a low-threshold laser device [2], [3], [4], [5], sensors [6], [7], [8], [9], reflective displays, deformable elastomers [10], [11], [12], [13], and camouflage technologies [14]. Using the intrinsic benefits of soft matter, where the molecular arrangements can be altered in a controllable manner, chiral photonic-band structures and a photonic stopband wavelength can be tuned by addressing various external stimuli such as light [15], [16], [17], [18], [19], [20], temperature [21], [22], [23], [24], [25], electric field [26], [27], [28], [29], [30], [31], [32], and mechanical deformation [33], [34], [35], [36].

The electric wavelength control of the chiral photonic band is particularly desirable for practical electro-optical applications. From this technical perspective, the research challenges [37], [38], [39], [40], [41] include obtaining the electrical controllability of chiral photonic systems. However, spirally rotated chiral structures are vulnerable to disruption and can easily lose their well-organized photonic-band periodicity under electric field stress [42]. Therefore, the photonic wavelength control of low molecular weight mesogenic chiral photonic structure systems is limited in specific conditions, e.g., when using a negative dielectric host with commanded surfaces [40] or ferroelectric electro-active dopants [41] to avoid the electric instability of a chiral photonic structure under direct electric fields. Approaches using helix oblique method, have been attempted. However, this method requires special chiral materials using chemically synthesized bi-mesogenic molecules with carefully designed molecular structure and finely controlled elastic constants. Furthermore, conventional low molecular weight mesogenic chiral photonic-band structures are also affected by mechanical deformations and can easily be disrupted. Therefore, a reliable photonic wavelength control method that can provide electric control and stability during wavelength changes in chiral photonic-band structures requires further research for practical implementation, particularly for flexible electro-optical applications. Shortly, direct electric wavelength control using conventional mesogenic materials is still challengeable.

On the other hand, when chiral photonic-band structures are templated with rubber-like mesogenic elastomers, the so-called chiral photonic gels (CPGs) can be obtained [35, 43, 44]. Because of their improved elastic properties, mechanically controlled stretchable CPGs provide considerable potential for broadband wavelength tunability with enhanced optical chiral stability.

In contrast to a low molecular weight mesogenic structure, mechanically stable helix deformations allow subsequent chiral pitch-length changes and broadband wavelength shifts from conventional chiral reactive mesogenic materials by addressing the mechanical stress-strain perpendicular to the standing helix of CPGs. Using this benefit of CPGs, the stable mechanical controls of broadband wavelength shifts in one-dimensional [43, 45] and three-dimensional chiral structures (the so-called stretchable blue-phase chiral liquid crystals) can be obtained [44]. However, the desirable electric wavelength control in CPGs also presents limitations. In general, the electric wavelength control of stretchable chiral photonic-band structure has not been established and requires further research.

In this study, we report for the first time a research approach using an electric photonic wavelength control of stretchable chiral photonic-band structure. Using a hybrid structure comprising CPGs on an electrically controllable soft actuator, electrically stretchable CPGs were created. Electrically stretchable CPGs with broadband wavelength tuning against electrical stress and mechanical deformation were demonstrated. In addition to electrically stretchable wavelength-tuning stability, the tuning reliability was confirmed via the repeated switching of the electrical stretching of reversible wavelength changes for up to 100 cycles. Similar to the full visible wavelength control of CPGs with mechanical stretching, the electrically stretched wavelength changes of CPGs were observed over a broadband wavelength ranging from 695 to 524 nm. Consequently, for the first time, ∼171 nm-sized wavelength changes with good electro-mechano-optical stability were obtained.

## 2 Results and discussion

**Figure 1:**
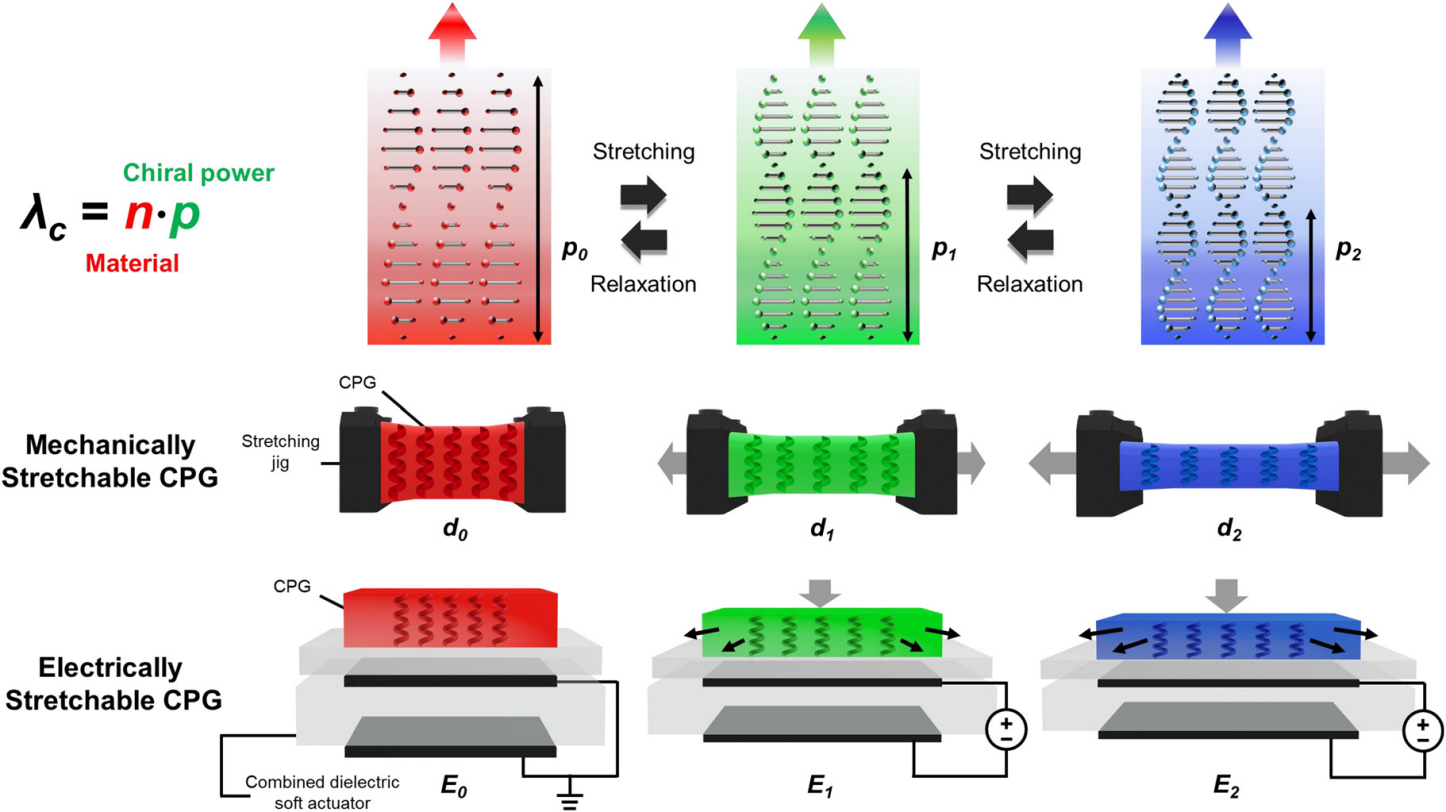
A schematic showing the photonic stopband wavelength-tuning mechanisms of electrically stretchable CPGs.

Figure 1 shows a schematic representation of the photonic stopband wavelength-tuning mechanisms of electrically stretchable CPGs. Following the de Vries condition [46], which is expressed as *λ*_c_ = *n*_avg_⋅*p*, the central wavelength of chiral photonic-band gaps (*λ*_c_) could be controlled using the average refractive index (*n*_avg_) and chiral repeating pitch length (*p*). In the case of a lateral strain, which typically manifests as deformation stress occurring perpendicular to the standing helix, the CPGs should be stretched alongside a tightening of the chiral pitch in accordance with a blue shift in the predefined photonic wavelength. To obtain electric control of stretching during wavelength changes in the chiral photonic system, a carefully prepared free-standing film of CPGs was integrated onto the dielectric elastomer soft actuator to impart a lateral stretching–relaxing strain between the top and bottom electrodes. For this electrical stretching controllability, a combined dielectric soft actuator was prepared using a 200% biaxially prestretched dielectric elastomer (VHB4905, 3M) actuator [47], [48], [49] with supporting polydimethylsiloxane (PDMS) (Sylgard184, Dow; see the Supplementary material). Note that the initial photonic-band condition of the CPGs was prepared with a central stopband at 695 nm with red-colored photonic reflection and enhanced elastic properties. Consequently, following the stable helix deformations with pitch tightening, a continuous color shift was allowed by either simple mechanical stretching or further electrical stretching methods. The evolutional mechano-optical and electro-mechano-optical wavelength behaviors were investigated by either direct mechanical stretching stress or via electrical stretching stress affected by hybrid CPGs.

**Figure 2:**
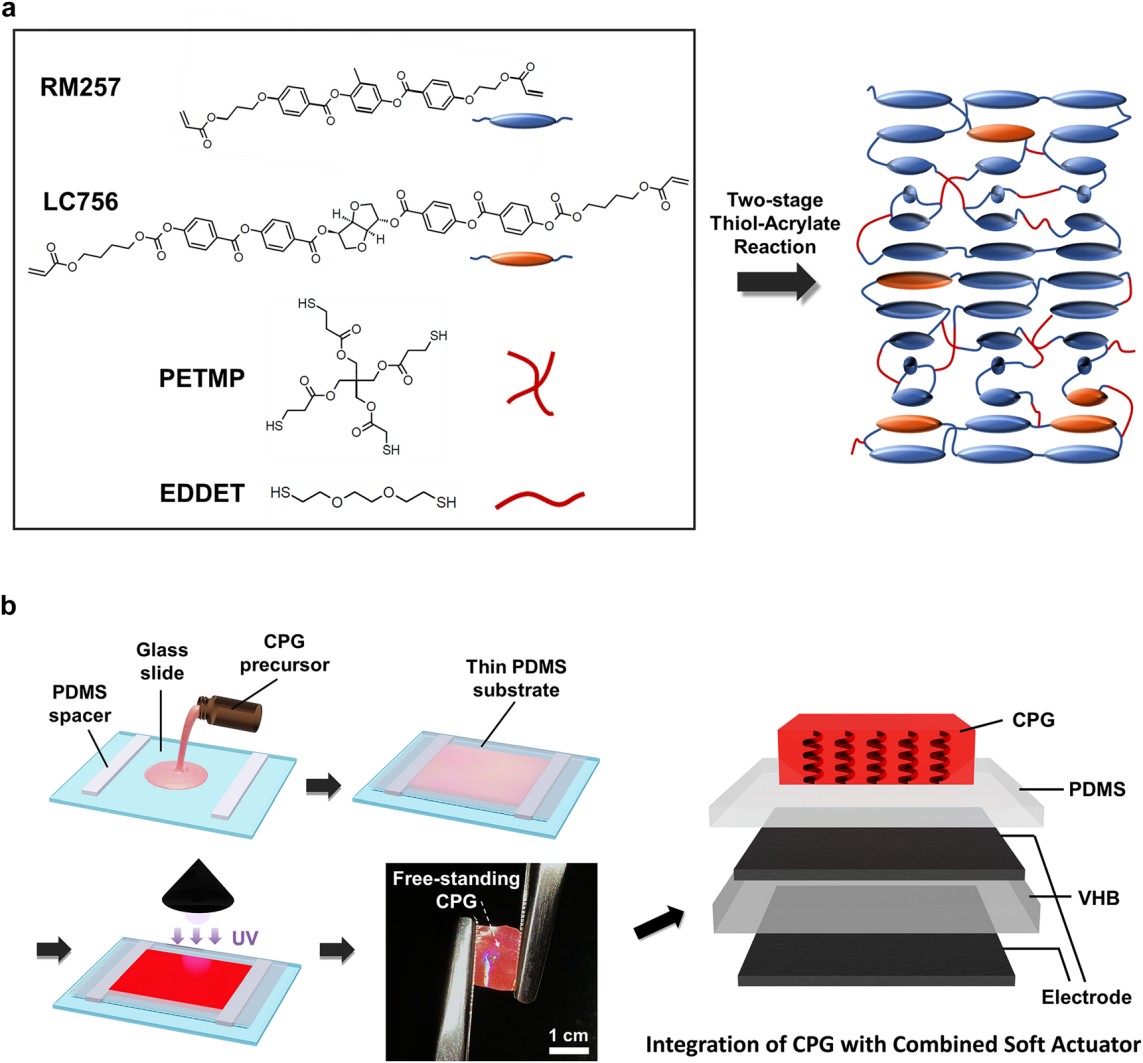
The preparation of mechanically and electrically stretchable chiral photonic gel samples: (a) description of curable chiral premixture materials; (b) preparation of integrated chiral photonic gels with a combined soft actuator.

Prior to investigating the stretching properties of chiral gels, an elastic CPG sample should be prepared with sufficient stability under stretching deformation stress conditions (either for mechanical stretching deformation or electrical stretching stress process, described in Figure 2). To establish the stretching robustness, an improved elastic CPG was prepared following a two-stage thiol-acrylate reaction [50], as shown in Figure 2(a). First, a curable chiral premixture of 4.1 wt% the chiral reactive mesogenic dopant (LC756, BASF) in the achiral diacrylate reactive mesogenic host material (RM257, GRANDINCHEM). The chiral premixture was designed to have a ∼420 nm pitch length and a red photonic reflective color before conducting additional wavelength-tuning processes. Then, 50 wt% of the chiral premixture was dissolved in toluene solvent for film casting. The final elastic CPG precursor preparation was completed by adding a di-thiol monomer 2,2′-(ethylenedioxy)diethanethiol (EDDET, Sigma-aldrich) additive and a tetra-functional thiol crosslinking monomer, i.e., pentaerythritol tetrakis(3-mercaptopropionate) (PETMP, Sigma-aldrich) as flexible spacer groups to functionalize the rubber-like elastic properties of chiral gels. The chemical structures and synthetic mechanisms of the precursor materials of the chiral gels are described in Figure 2(a). The prepared final chiral precursor solution was cast on a bottom glass substrate and a 500 μm thin PDMS top-cover with a 100 μm thickness spacing from the bottom substrate. Subsequently, a uniform solid chiral precursor film was obtained after slow solvent evaporation for ∼24 h at room temperature in the dark. At the end of the synthetic sample creation process, the free-standing CPGs were prepared by photo-polymerization at *λ* = 365 nm using a 30 mW cm^−2^ light source for 10 min in an ultraviolet oven [CSM1010, AUVCURE; see Figure 2(b)]. The intended red photonic color reflection with alignment quality was confirmed from spectral and microscopic texture observations.

**Figure 3:**
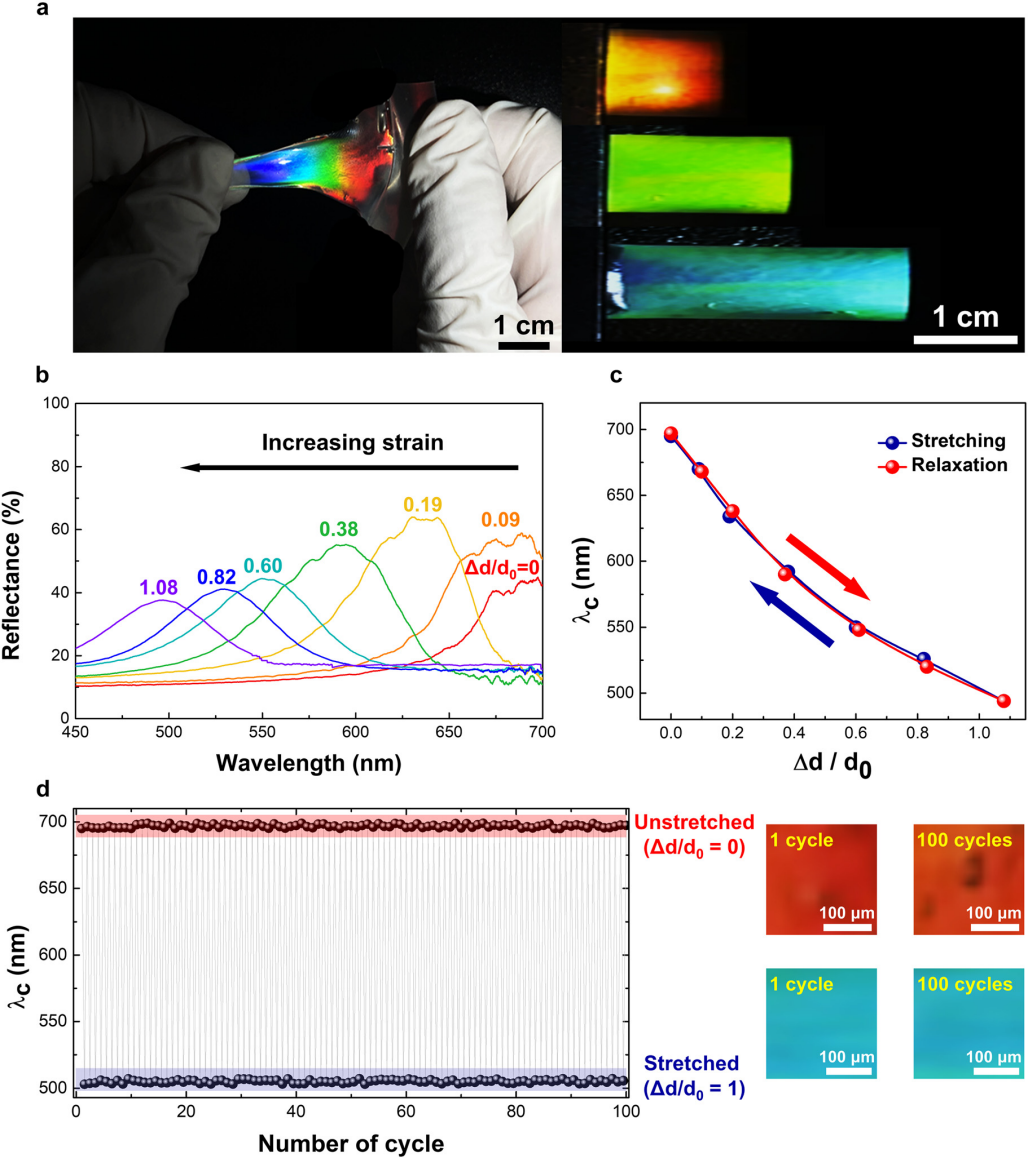
Mechano-optical changes in the properties of CPGs: (a) reflective color change in CPG samples by mechanical stretching; (b) photonic wavelength blue-shift as a function of the addressed mechanical strain; (c) reversible central photonic wavelength (*λ*_c_) changes as a function of stretch power (Δ*d*/*d*_0_, where ‘Δ*d*’ is the stretched scale of length and ‘*d*_0_’ is the initial sample length); and (d) repeatable and recovered wavelength control of mechanically stretched CPGs up to 100 stretching–relaxation iteration cycles with 100% further stretching of CPGs.

To examine the optical stretching properties of CPGs, highly elastic free-standing CPGs were attached to the dielectric elastomer soft actuator, including compliant top and bottom electrodes and supporting PDMS film. Before conducting the electrical stretching study, the final CPG samples were stretched on the elastic soft actuator and the subsequent mechano-optical changes in the properties of CPGs were investigated, as shown in Figure 3.

When observing the mechanical stress–strain, a vivid photonic reflective color change from red to blue was detected from the prepared stretchable CPGs (see Figure 3(a) and the Supplementary material, Video S1). The chiral photonic-band wavelength change was monitored using an in-house measurement setup that included a spectroscope (Flame-T, Ocean Optics), complementary metal-oxide-semiconductor image sensor (HAWK-SCM63, Zootos), and a carefully designed in-house stretching jig on a modified microscope (BX51, Olympus) system. A mechano-optical blue-shifted photonic wavelength was observed from *λ*_c_ = 695 to *λ*_c_ = 494 nm toward a shorter wavelength as a function of stretch strain (Δ*d*/*d*_0_, *λ*_c_) as Figure 3(b). Furthermore, the stretching-induced wavelength shifts in the central photonic-band wavelength (*λ*_c_) as a function of stretch power, which is denoted as “Δ*d*/*d*_0_” (where Δ*d* is the stretched length scale and *d*_0_ is the initial sample length) was entirely reversible and continuous [see Figure 3(c)]. Moreover, the stretching deformation induced wavelength switching was fully repeatable against 100 stretching–relaxation iterations employing the stretching condition of Δ*d/d*_0_ = 1 [see Figure 3(d)]. The mechano-optical stretching wavelength change was stable without a significant disruption of the chiral photonic structure or hysteresis of photonic wavelength positions during the tuning process.

**Video S1:** 

**Figure 4:**
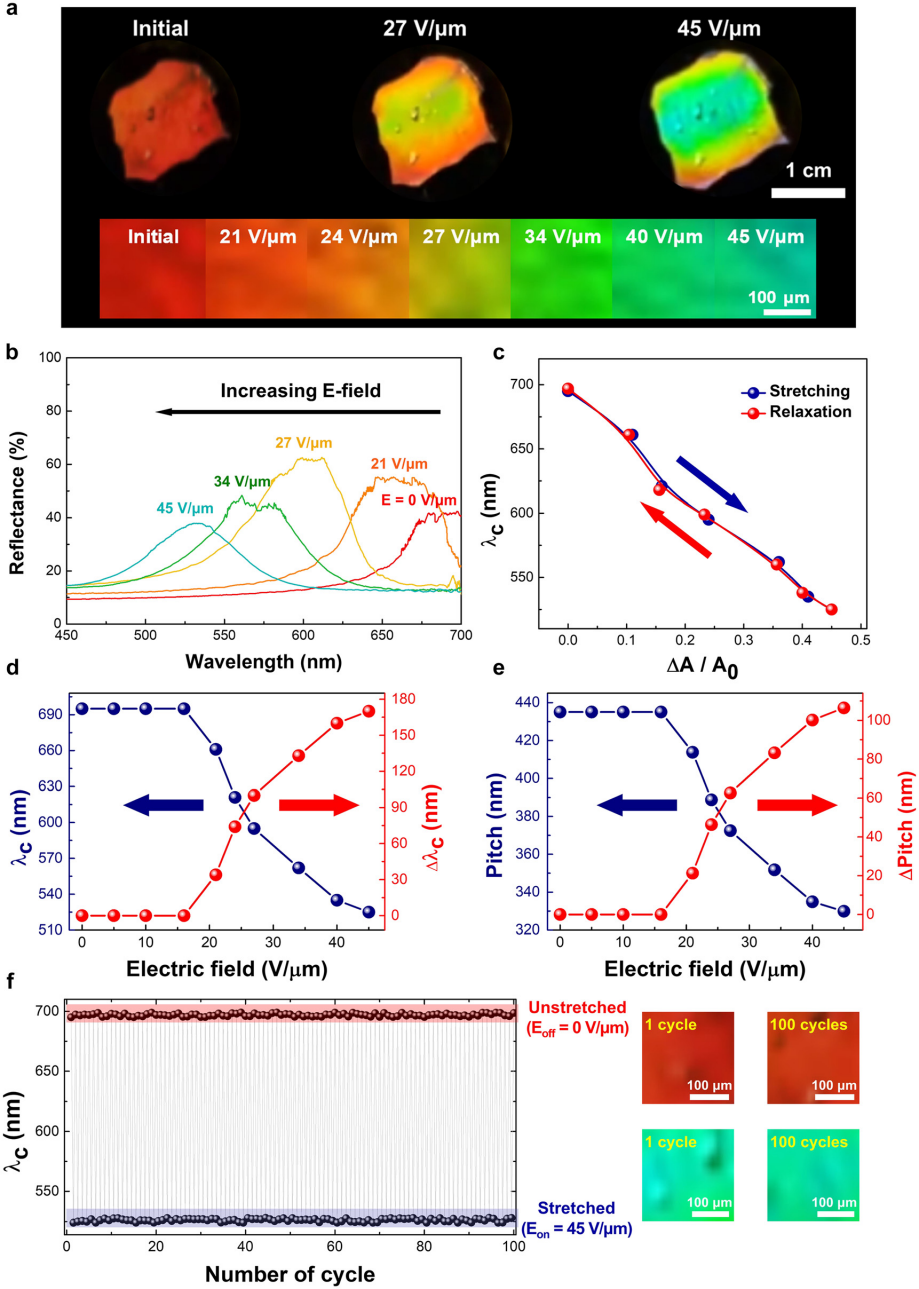
Electro-mechano-optical changes in the properties of chiral photonic gels: (a) reflective color change in electrically stretched chiral photonic gels as a function of the applied electric field; (b) photonic wavelength blue-shift of electrically stretched chiral photonic gels as a function of the applied electric field strength; (c) reversible central photonic wavelength (λ_c_) changes as a function of stretch power (ΔA/A_0_, where ‘ΔA’ is the stretched scale of area, and ‘A_0_’ is the initial sample area of electrode); (d) reversible central photonic wavelength (λ_c_) positions and electrically controlled wavelength changes as a function of the applied electric field strength; (e) calculated chiral pitch length and electrically controlled pitch changes as a function of the applied electric field strength; (f) repeatable and recovered wavelength control of electrically stretched chiral photonic gels up to 100 times from continuous, repeating electrical E-on and E-off-switching of chiral photonic gels with electrical stretching conditions of 45 V/μm. Notably, the electrical stimulus response of VHB in the combined soft actuator induced an indirect strain of chiral photonic gels. The frequency of the applied electric field was 0.2 Hz sine wave.

Electric stretching-induced electro-mechano-optical photonic behavior changes in the CPGs were investigated by the electrical triggering of the dielectric soft actuator on which the CPGs had been placed (see Figure 4). To investigate similar optical stretching observations with electrical actuation, the microscopic measurements obtained using a spectrometer was slightly modified by removing the mechanical stretching jig and connecting an electrical signaling source, which comprised a function generator (AFG1022, Tektronix), an oscilloscope (TBS2000B, Tektronix), and a high-voltage amplifier (609B-3, Trek). When applying an electric field to the dielectric soft actuator, a vivid photonic reflective color shift from red color to deep green was clearly observed for the electro-elastic CPGs on the dielectric soft actuator as a function of the applied electric field (see Figure 4(a) and the Supplementary material, Video S2). Note that the electrical stretching photonic change was stable under electrical stress, and mechanical deformation/scattering haze typical of conventional chiral photonic-band structure disruption was not observed. Considering the electrical areal expansion behavior of the dielectric soft actuator with the vertical electric field between the compliant top and bottom electrodes, the laterally expanding stretching power (Δ*A*/*A*_0_) resulted in the stable and continuous electro-mechanical stretching of CPGs (see Supplementary material, Figure S1).

**Video S2:** 

In further, the electro-mechano-optical stretching wavelength changes in CPGs on an electrically signaled soft actuator were also monitored [see Figure 4(b)]. From the investigation of laterally extending electric stretching power, denoted as Δ*A*/*A*_0_, (Supplementary material, Figure S1), similar observation of wavelength change in terms of central chiral photonic-band wavelength (*λ*_c_) as a function of electrical stretching power (Δ*A*/*A*_0_) was studied in Figure 4(c). The central chiral photonic-band wavelength (*λ*_c_) had observably blue-shifted toward a shorter wavelength as a function of the electric field [695 nm at 0 V/μm, 621 nm at 24 V/μm, and 524 nm at 45 V/μm; see Figures 4(b) and (d)]. The maximum electrically stretchable tuning shift of wavelength change (Δ*λ*_c_) was ∼171 nm from 695 nm with 0 V/μm up to 524 nm with 45 V/μm. Moreover, the sufficient electric field strength required to overcome the threshold electric field was ∼15 V/μm because of the elastic storing energy of dielectric soft actuator and CPGs.

The above electro-mechano-optical wavelength changes covered a significant range of visible wavelength bands. Furthermore, this is the highest tuning record to date of chiral photonic wavelength control using an electrical method with mechanical stability. The tuned chiral photonic wavelength position of CPGs was stable and fully recovered similar to the direct mechanical stretching case shown in Figure 3. Compared with the direct mechanical stretching results, the maximum tuned wavelength change was relatively smaller, possibly caused by the relatively weak maximum lateral stretching power, denoted as Δ*A*/*A*_0_, a maximum of ∼50% of the soft actuator compared with the stretching power in terms of Δ*d*/*d*_0_, with the maximum >100% by the motorized stretching jig (Figure 3(c)), (c) as described in the Supplementary material (Figure S1). However, the effective wavelength change (Δ*λ*_c_) for the same dimensional change (Δ*A*/*A*_*0*_, Δ*d*/*d*_0_) was stronger in electrically isotropic strain power (Δ*A*/*A*_0_) (Figure 4(c)) than mechanical uniaxial strain power (Δ*d*/*d*_0_) (Figure 3(c)). For example, unlike the wavelength change (Δ*λ*_c_) of ∼103 nm in Δ*d*/*d*_0_ ∼40% condition in mechanically uniaxial strain in Figure 3(c), the wavelength change (Δ*λ*_c_) of ∼160 nm was observed in Δ*A*/*A*_0_ ∼40% condition in electric stretching isotropic strain in Figure 4(c). This could be because the 2D isotropic strain in the electrical stretching condition was stronger and more effective than that in the one-dimensional uniaxial strain by mechanical stretching. The calculated chiral pitch-length tightening amount in terms of pitch change (Δ*p*), based on the de Vries’ condition and function of the electric field, ranged from 414 to 337 nm. The resulting Δ*p* behavior was similar to the wavelength changes (Δ*λ*_c_) as a function of the electric field [see Figure 4(d) and (e)]. The wavelength switching via electrical signal was fully repeatable against 100 stretching–relaxation iterations with a stretching condition of *E* = 45 V/μm [see Figure 4(f)]. Overall, the results indicated that the electrical wavelength control of CPGs was stable, reversible, and occurred in a continuously similar to the mechano-optical wavelength control shown in Figure 3.

**Figure 5:**
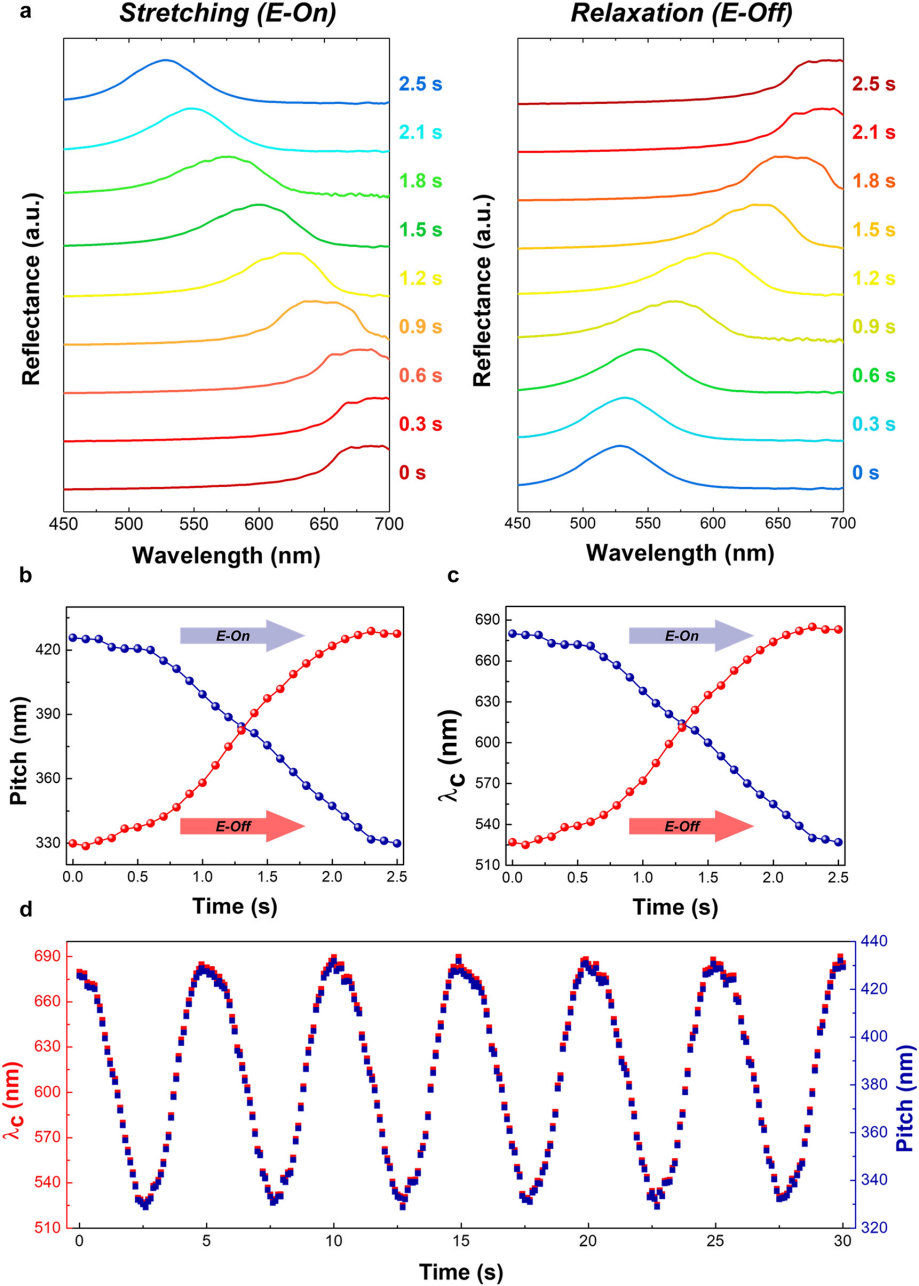
Time-tracked electro-mechano-optical wavelength changes in the properties of CPGs: (a) evolutional photonic wavelength shifts of electrically stretchable CPGs in a state between applying (E-On, stretching) and removing (E-Off, relaxation) an electric field of 45 V/μm at a 0.2 Hz sine wave signaling condition; (b) symmetrical reversible switching of the central photonic wavelength (*λ*_c_) position; (c) reversible chiral pitch-length changes between E-On (stretching) and E-Off (relaxation) of a 45 V/μm electric field at a 0.2 Hz sine wave; (d) time-based monitoring of electrically stretchable CPG switching in terms of central photonic wavelength (*λ*_c_) position tracking and tuned pitch length (*p*) during a continuously applied electric field of 45 V/μm at a 0.2 Hz sine wave for up to 30 s.

Additional electro-mechanic stretching properties were evaluated by time-based monitoring of the evolutional shifts in the electrically stretched CPGs (see Figure 5). The vivid but continuous blue-shift at photonic wavelength changes in a 45 V/μm electric field with a 0.2 Hz sine wave frequency was observed as a function of time [see Figure 5(a)]. Moreover, the recovery of the central chiral photonic wavelength position and chiral pitch length altering at the removing electric field (E-Off) state was almost equal to the tuning process at the applying electric field (E-On) state [see Figure 5(b) and (c)]. Additionally, further consideration of the time tracking of the central chiral photonic wavelength (*λ*_c_) positions in the electrical stretching and relaxation processes at E-On (stretching) and E-Off (relaxation) showed that the controlled wavelength changes of CPGs were continuously stable and symmetrically reversible. Therefore, the pitch tightening change process of the helicoidal chiral structure occurred gently and excluded any sudden pitch jumps [see Figure 5(d)]. The sudden pitch jumps and return to a longer wavelength during pitch tightening are common practical aspects of the chiral pitch tightening process [1, 51, 52] and technical huddle. Consequently, the electrically stretchable wavelength control, mechanical stability, and continuous broadband wavelength changes in a chiral photonic-band structure were confirmed in this study for the first time.

## 3 Conclusions

In this study, an electro-mechano-optical system of electrically stretchable CPG was investigated for the first time. By circumventing the chiral photonic-band structure disruption under an electric field and mechanical deformations, a stable electric control of phonic wavelength shift exploiting an elastic CPG on a dielectric soft actuator was presented. The electrically expanding actuation of the soft actuator successfully induced further stretching deformations on the standing CPGs. As a result, fully visible photonic wavelength control with repeated switching in a chiral photonic-band liquid crystal system was obtained. Moreover, a stable and electrically stretchable broadband wavelength change up to 171 nm of the central photonic-band wavelength change (Δ*λ*_c_) was observed in a chiral liquid crystal photonic-band structure for the first time. The electrically stretchable CPG system was fully reversible and repeatable for up to ∼100 iterations. The discovered CPGs could be applied to various practical tunable photonic applications using the reliable photonic broadband wavelength control with an electric signaling strain method and spontaneous reproduction of a chiral photonic-band structure.

## Supplementary Material

Supplementary Material Details

Supplementary Material Details

Supplementary Material Details

## Supplementary Material

Technical supporting information are prepared in separated enclosed information with supplemental video.
