# Supplementary material for: Broadband wavelength tuning of electrically stretchable chiral photonic gel
Source: Nanophotonics. 2022 Jan 4;11(9):2139–48. doi: 10.1515/nanoph-2021-0645 (PMC11501980; doi:10.1515/nanoph-2021-0645)
Supplement: Supplementary file 5 — Supplementary Material Details [file j_nanoph-2021-0645_suppl_001.docx]

**Supplementary Information**

Seungmin Nam, Dahee Wang, Gyubin Lee, and Su Seok Choi*

Broadband Wavelength Control of Electrically Stretchable Chiral Photonic Gels

Department of Electrical Engineering, Pohang University of Science and Technology (POSTECH), Pohang, 37673, Korea

*Corresponding E-mail: choiss@postech.ac.kr (Prof. Su Seok Choi)

**1. Synthesis of chiral photonic gels**

Mechanically or electrically stretchable highly elastic chiral photonic gels, including a reactive mesogenic host and a thiol chain extender, were synthesized using a two-stage thiol-acrylate reaction following the process in [1, 2]. First, a chiral premixture of 4.1 wt% chiral reactive mesogenic dopant (3R,3aS,6aS)-hexahydrofuro[3,2-bfuran-3,6-diyl bis(4-(4-((4-(acryloyloxy) butoxy) carbonyloxy) benzoyloxy) benzoate) (LC756, BASF) in the achiral diacrylate reactive mesogenic host 1,4-bis-[4-(3-acryloyloxypropyloxy) benzoyloxy]-2-methylbenzene (RM257, GRANDINCHEM) was prepared. Then, the prepared chiral premixture was dissolved in toluene with a 50 wt% concentration at 80°C for 10 min. A final precursor of chiral photonic gels was prepared by adding thiol chain extenders (di-thiol monomer 2,2′-(ethylenedioxy)diethanethiol (EDDET), tetra-functional thiol crosslinking monomer pentaerythritol tetrakis(3-mercaptopropionate) (PETMP), and photoinitiator (2,2-dimethoxy-2-phenylacetophenone (Irgacure 651, Sigma Aldrich)) to the prepared chiral premixture solution. Finally, a diluted catalyst solution comprising dipropylamine (DPA, Sigma Aldrich) and toluene at a weight ratio of 1:50 was added to the solution and vigorously stirred for 5 min in preparation for film casting. The molar ratio of RM257/EEDET/PETMP/Irgacure 651/DPA in the final precursor solution for chiral photonic gels was 1/1/0.012/0.016/0.02). Table S1 shows the details regarding the accurate amounts of each material component for creating chiral photonic gels.


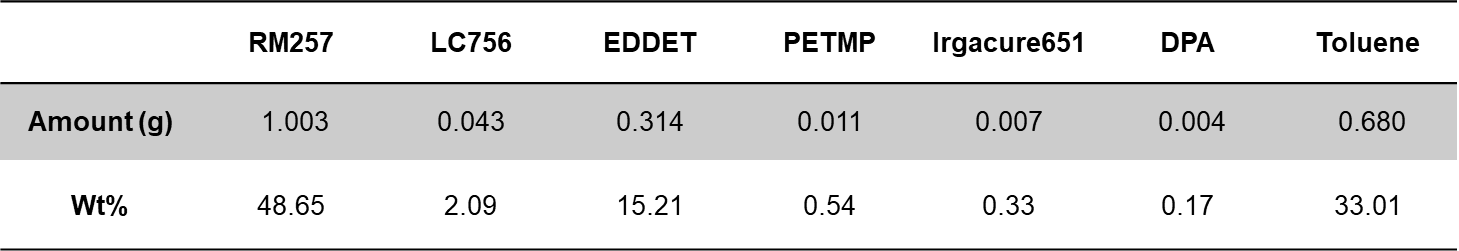


Table S1. The amounts and wt% of materials used for preparing the chiral photonic gels precursor.

**2. Fabrication of the dielectric soft actuator**

To achieve electrically triggered stretching control, a combined dielectric soft actuator was prepared. First, a commercial dielectric adhesive film (VHB 4905 film, 3M) was prestretched with a radial strain of 300% and sandwiched with compliant top and bottom electrodes using carbon conductive grease (MG Chemicals). A rigid frame was placed on the prestretched film to maintain the prestretched elastic actuating power of the VHB film. Finally, the dielectric soft actuator was completed by placing a thin (50 μm) supporting layer on the prestretched VHB film. The supporting layer was placed by spin coating a polydimethylsiloxane (PDMS; Dow, Sylgard184) silicone elastomer base at a 30:1 ratio to a curing agent at 2,000 rpm for 30 s above the VHB film. Moreover, a small amount of the precursor of PDMS was dropped on the central part of the actuator where the CPG should be mounted. After placing the prepared free-standing CPG on the uncured PDMS surface of the PDMS/VHB coupled actuator, the surface PDMS precursor interface was thermally cured at 60 °C for 2 h to obtain an excellent adhesive interface and retain the CPG on the combined actuator.The electrical stretching power of the fabricated dielectric soft actuator was examined by monitoring actuating active aerial expansion. This process employed an integrated setup with laser displacement measurements (LJ-V7200, Keyence), a function generator (AFG1022, Tektronix), an oscilloscope (TBS2000B, Tektronix), and a high-voltage amplifier (609B-3, Trek). The stretching power of dielectric soft actuator was increased up to ΔA/A_0_ = 0.5, where A_0_ and ΔA are the initial active area of the soft actuator and changes in the active area at the E-field on-state, respectively (Figure S1). Note that additional increase in the electrical stretching power was limited owing to the disruption of actuator films.


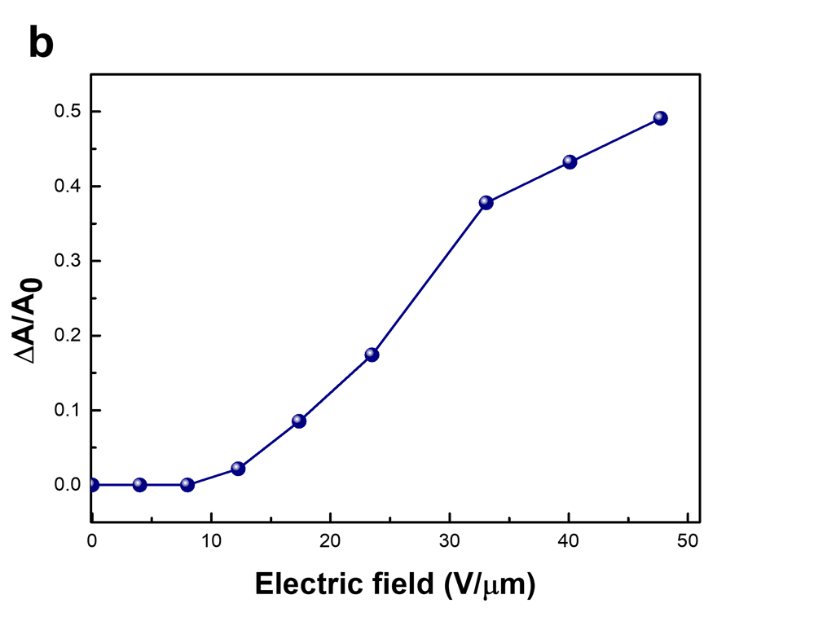


Figure S1. The electrical actuation performance of fabricated dielectric soft actuator as a function of the electric field.

**3. Supplementary videos**

Video S1
A video demonstrating the reflective color change and reversibility from red to blue with mechanical stretching.

Video S2
A video demonstrating the reflective color change and reversibility from red to deep-green with electrical stretching.

[1] Kizhakidathazhath R, Geng Y, Jampani VS, Charni C, Sharma A, Lagerwall JP. Facile anisotropic deswelling method for realizing large‐area cholesteric liquid crystal elastomers with uniform structural color and broad‐range mechanochromic response. Adv Funct Mater 2020, 30, 1909537.

[2] Saed MO, Torbati AH, Nair DP, Yakacki CM. Synthesis of programmable main-chain liquid-crystalline elastomers using a two-stage thiol-acrylate reaction. J Vis Exp 2016, 2016, 1-10.
