# Supplementary figures and images for: Broadband wavelength tuning of electrically stretchable chiral photonic gel
Source: Nanophotonics. 2022 Jan 4;11(9):2139–48. doi: 10.1515/nanoph-2021-0645 (PMC11501980; doi:10.1515/nanoph-2021-0645)

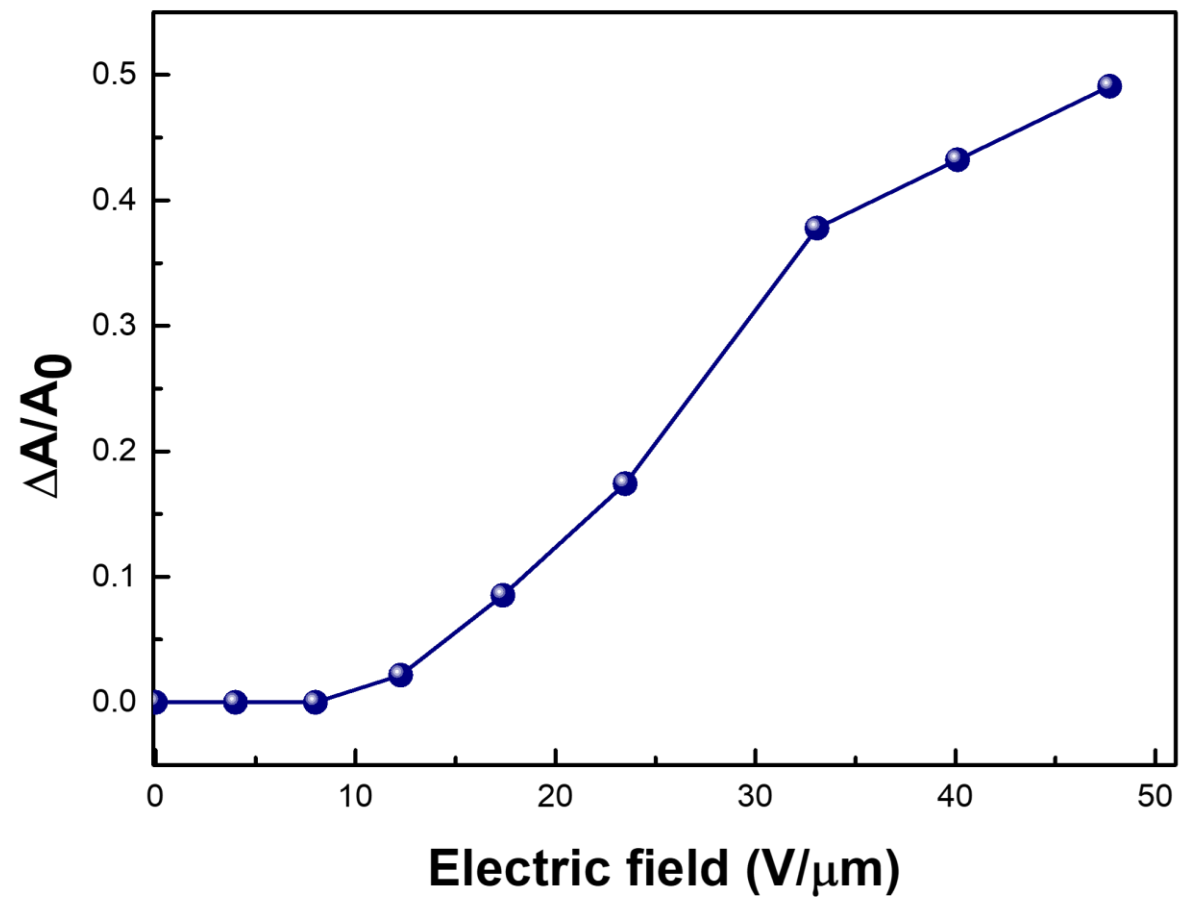

Supplement: Supplementary file 6 — Supplementary Material Details [file j_nanoph-2021-0645_suppl_002.pdf]
