# Supplementary material for: Broadband wavelength tuning of electrically stretchable chiral photonic gel
Source: Nanophotonics. 2022 Jan 4;11(9):2139–48. doi: 10.1515/nanoph-2021-0645 (PMC11501980; doi:10.1515/nanoph-2021-0645)
Supplement: Supplementary file 7 — Supplementary Material Details [file j_nanoph-2021-0645_suppl_003.pdf]

	RM257	LC756	EDDET	PETMP	Irgacure651	DPA	Toluene
**Amount (g)**	1.003	0.043	0.314	0.011	0.007	0.004	0.680
**Wt%**	48.65	2.09	15.21	0.54	0.33	0.17	33.01
